# Risk Factors for Overweight and Obesity within the Home Environment of Preschool Children in Sub-Saharan Africa: A Systematic Review

**DOI:** 10.3390/nu14091706

**Published:** 2022-04-20

**Authors:** Albert L. Kwansa, Robert Akparibo, Joanne E. Cecil, Gisele Infield Solar, Samantha J. Caton

**Affiliations:** 1 Public Health Section, School of Health and Related Research (ScHARR), The University of Sheffield, Sheffield S1 4DA, UK; alkwansa1@sheffield.ac.uk (A.L.K.); r.akparibo@sheffield.ac.uk (R.A.); 2 School of Medicine, Population and Behavioural Sciences, University of St Andrews, St Andrews KY16 9TF, UK; jc100@st-andrews.ac.uk; 3 School of Medicine, The University of Sheffield, Sheffield S10 2TN, UK; ginfieldsolar1@sheffield.ac.uk

**Keywords:** preschool, obesity, overweight, Sub-Saharan Africa, home environment

## Abstract

Sub-Saharan Africa (SSA) is experiencing an increasing prevalence of young children being overweight and obese. Many feeding and physical activity-related behaviours are established at home during preschool years, yet the precise factors that contribute to preschool overweight and obesity have not been fully elucidated. This review aims to identify factors in the home environment associated with overweight and or obesity in preschool children in SSA. Ovid MEDLINE, EMBASE, CINAHL, Scopus, Web of Science, Africa Journals Online (AJOL) and the African Index Medicus databases were systematically searched for qualitative and quantitative studies published between 2000 and 2021. Eleven studies (ten quantitative, one qualitative) met the inclusion criteria. Overall, the results highlight the paucity of studies exploring factors in the home environment associated with overweight and obesity in preschool children in Sub-Saharan Africa. The home food environment and maternal BMI appear to be important factors associated with overweight and obesity in preschool children; however, the information for all other factors explored remains unclear due to the lack of evidence. For successful obesity prevention and treatment interventions to be developed, more research in this area is required to understand how different aspects of the home environment contribute to overweight and obesity in preschool Sub-Saharan African children.

## 1. Introduction

The dual burden of malnutrition, defined as the co-existence of overweight, obesity and undernutrition, is an ever-increasing problem in adults and paediatric groups in developing countries at the individual level through to the population level [1]. Undernutrition is a persisting problem in low- and middle-income countries, and over the last decade, many nutrition programs have focused on undernutrition. However, obesity is now a problem in low- and middle-income countries due to rapid urbanisation and economic development [2]. According to the World Health Organization, in Africa the number of preschool children under the age of five who are overweight has increased by around 24% since 2000 [3]. Population-level data demonstrate that the combined prevalence of overweight and obesity in Sub-Saharan African preschool children is approximately 6.8% [4], a public health crisis that is expected to worsen in the absence of successful population-wide interventions and government policies.

Children with excess bodyweight are more likely to have excess bodyweight in adulthood [5,6] and therefore increased likelihood of developing associated noncommunicable diseases [7]. Moreover, evidence suggests that children who are overweight and obese are more likely to report psychological ill health and stigmatising behaviours compared with individuals of normal weight [8,9].

The aetiology of obesity is complex involving interaction between biological, social, environmental and economic factors [10]. The home environment, particularly in the early years, plays a key role in promoting or discouraging health-related behaviours in children and therefore provides a focal point for targeting interventions for overweight and obesity. Young children spend a large proportion of their time in the home, where they consume the majority of their meals [11,12]. Given that parents and caregivers are likely to share meals and engage in physical activity and screen-based sedentary activities together, children’s behaviours are often associated with behaviours of parents and caregivers [13,14,15,16,17].

In a recent systematic review, Kininmonth et al. [18] used a conceptual model of the “obesogenic” home environment originally proposed by Gattshall et al. [19] and modified by Schrempft et al. [20] to examine the relationship between the home environment and adiposity in children less than 12 years old. Their findings demonstrated that the home media environment, notably greater access to electronic devices, was most consistently associated with excess adiposity in children, with less consistent findings for the home food and physical activity environments.

**Figure 1:**
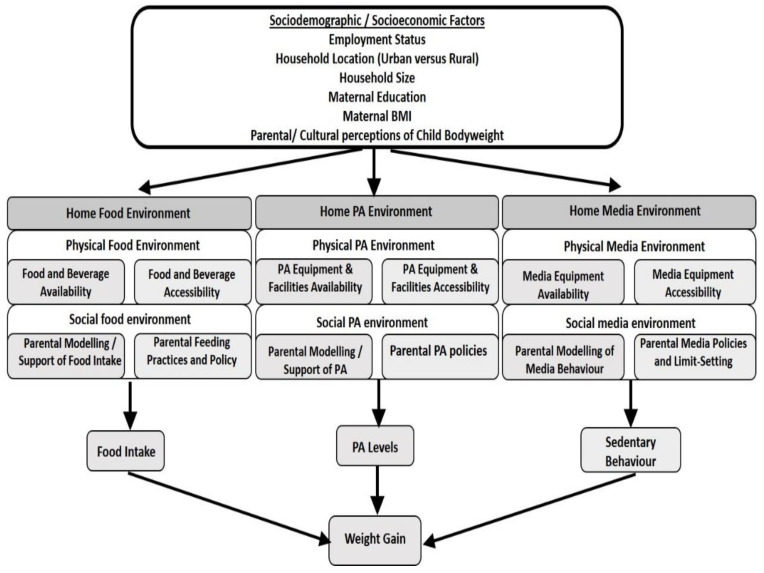
Adapted conceptual model of the obesogenic home environment [20]. Added components: Sociodemographic and socioeconomic factors.

For the purpose of the current review, we further expanded this conceptual model of the “obesogenic home environment” [20] to include other potential important socioeconomic and sociodemographic influences on energy balance (Figure 1). Socioeconomic and sociodemographic factors such as household income and wealth status, maternal education and maternal BMI are factors with the capacity to influence food intake, physical activity and sedentary behaviours [20,21]. In higher-income countries, social gradients are observed for weight status and diet quality, with less affluent populations being more predisposed to developing overweight and obesity and consuming less nutrient-dense and more energy-dense diets compared with higher-income populations [22,23,24,25]. Similar patterns for physical activity [26] and sedentary screen time activity in children were also observed [14]. Importantly, mothers are often the primary caretakers of children in SSA and are responsible for the purchasing, preparation and allocation of food in the home environment [27]. Maternal characteristics such as maternal education [28,29] and household demographics such as household size were previously associated with poor infant and young child feeding practices in Africa [30], with the potential for these to extend throughout the preschool years. Further, cultural beliefs surrounding feeding practices and perceived child size are also implicated in infant and child feeding [31]. A recent systematic review and meta-analysis, including studies from 79 international settings reported significantly increased odds of childhood overweight or obesity for mothers who were overweight and obese [32].

It is currently unknown to what extent the home environment is associated with overweight and obesity in preschoolers in SSA, thus hindering the development of successful interventions for the prevention and treatment of paediatric excess adiposity. Therefore, the aim of this systematic review is to explore which aspects of the modified obesogenic home environment model are associated with overweight and obesity in preschool children in SSA.

## 2. Materials and Methods

The review was conducted according to the Preferred Reporting Items for Systematic Review and Meta-Analysis Protocols (PRISMA-P) 2020 guidelines [33] and was registered with PROSPERO (CRD42020220314). A mixed-methods approach was used to gather and summarise the evidence (qualitative and quantitative) for this review. This method was preferred for its usefulness and unique value in providing the completeness and contextual understanding of complex topics in public health studies [34,35], such as those relating to the home environment of preschool children. An adapted and expanded version of the home environment model [20] was employed to guide reporting of the study outcomes.

### 2.1. Literature Search

An initial search strategy was developed to scope the extent of the available literature and to identify synonyms related to the population (preschoolers in SSA) and outcomes (overweight and obesity) of interest. By using that initial search strategy, a scoping search was conducted in Ovid MEDLINE (Supplementary Materials). The results of that scoping search were used to test and refine the search strategy for a comprehensive search of the literature in seven electronic databases (Ovid MEDLINE, EMBASE, CINAHL, Scopus, Web of Science, Africa Journals Online (AJOL) and the African Index Medicus). A complete list of search terms for all databases is provided in the supplementary information (Supplementary Materials) for this review. Search terms in the search strategy included those related to preschool, overweight, obesity and Sub-Saharan Africa, and combined with Boolean operators for the literature published in English between 1 January 2000 and 31 December 2021. It was decided a priori to restrict the search to studies published from 2000 to reflect the increase in the prevalence of child obesity in Africa from that time [36,37,38]. Searches were conducted in March 2022. The reference lists of studies that were selected were also screened for the other potentially relevant literature that may have been missed. The outputs of the comprehensive searches were transported into Mendeley (version 1.19.5) for deduplication.

### 2.2. Selection of Studies

Studies identified from the literature search were screened to ascertain their relevance after duplicates were removed. Only published and accessible studies/papers were considered. Studies were first filtered for relevance by one reviewer (ALK), by title and then by abstract, in accordance with inclusion/exclusion criteria (Table 1). Studies focusing on preschool children aged two to six years old were selected for inclusion. At around the age of two years, children become more independent eaters, are more likely to display food neophobia [39], become more food fussy [40] and begin to respond to external food cues in the environment [41,42], compared with younger children. Due to potential differences in eating behaviours, studies focusing on children younger than two years old were excluded from the current review. Studies were included if age-specific data could be extracted. Key primary outcomes are factors in the modified obesogenic home environment model (Figure 1) that are associated with overweight/obesity in preschool sub-Sharan African children. A sample of relevant abstracts (10%) was independently reviewed and verified by two authors (SJC, RA) to eliminate selection bias. Three authors (ALK, RA, JC) independently screened all potentially relevant full texts, and the final decision on article inclusion/exclusion was made by consensus. Any discrepancies were overseen by a fourth reviewer (SJC).

### 2.3. Assessment of Study Quality

The quality of studies was independently assessed and collectively verified by three authors (ALK, RA, JC), using the Joanna Briggs Institute (JBI) critical appraisal checklists for cross-sectional [43] and qualitative studies [44]. The JBI checklist for cross-sectional studies assesses study quality based on eight criteria, including bias, confounding, the validity of measurement of exposures and the outcome and the validity of methods of analyses. The checklist for qualitative studies assesses study quality based on 10 criteria, including the congruity of philosophical perspective, congruity of research methodology with different aspects of the data and research question, researcher influence on study outcome and analysis approaches. For this review, each criterion was assigned a score of 1, with a maximum score of 8 indicating the highest quality of evidence for quantitative studies, 3–5 for medium quality and 0–2 for low quality; for qualitative studies, a score of 8–10 represented a high-quality study, 5–7 for medium-quality studies and 0–4 for low-quality studies.

### 2.4. Data Extraction

The main outcome of all included primary studies was overweight or obesity among SSA preschoolers. Data were extracted from the studies that met the review inclusion criteria. ALK used a data extraction form to extract key data, which was verified by a second reviewer (SJC). Relevant information extracted was the publication year, author(s), country of origin of the study, study design, population, setting, methods and key findings.

### 2.5. Data Synthesis

Data were analysed by first grouping all home environment variables into themes, guided by the home environment framework (Figure 1). These themes represented the physical and social aspects of the home food environment, the home physical activity environment and the home screen/media environment, as well as other relevant measures of the home environment that were adopted for this review. After grouping these measures of exposure, the direction of the evidence relating to the home environment and obesity/overweight among SSA preschoolers was examined based on the results or main findings from the included primary studies.

## 3. Results

**Figure 2:**
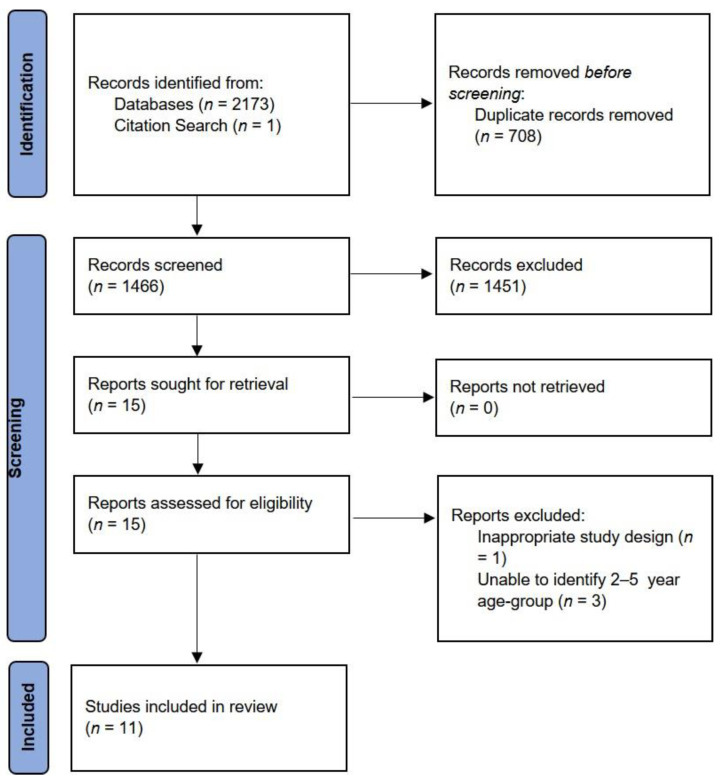
PRISMA Flow Diagram Showing the Process of Study Selection [33].

The search yielded a total of 2174 studies. Seven hundred and eight duplicates were removed (Figure 2). Title and abstract screening of the remaining 1466 studies further eliminated 1451 studies. Thus, 15 full-text papers were assessed for eligibility. Three studies were further excluded for not meeting the age requirements for the study population [4,45,46] and one for not having an appropriate study design [47]. A total of 11 full-text studies, comprising 10 quantitative studies and 1 qualitative study, were included in this review (Table 2 and Table 3).

### 3.1. Study Characteristics

The included studies were published between 2005 and 2020. The eleven studies that were found were conducted in six Sub-Saharan African countries (Ethiopia—3 [48,49,50], Kenya—2 [51,52], Nigeria—2 [53,54], South Africa—2 [55,56], Cameroon—1 [57] and Ghana—1 [58]). The 11 studies included 10 cross-sectional quantitative studies [48,49,50,51,52,53,54,55,57,58] and one qualitative study [56]. Overall, all studies contributed 4857 participants, with the sample size of individual studies ranging from 16 to 1495. The qualitative study included parents of preschoolers aged 3–5 years [56]. Six of the eleven studies included in the review measured child dietary intake within the home environment [48,49,50,53,57,58]. Three of the eleven studies included in this review explored the home physical activity environment [48,50,58], and three studies explored the home media environment [48,52,53]. With regards to socioeconomic and sociodemographic variables, three studies [51,57,58] examined the relationship between the BMI of mothers and overweight and obesity. Six studies reported on compositely measured household wealth index or SES scores based solely on the possession of pre-specified household assets [48,50,51,54,57,58], while two studies reported on ownership of specific assets such as TVs or computers [52] or a car [49]. Eight studies investigated maternal education [48,49,50,51,52,55,57,58]. Six studies explored the employment status of mothers/caregivers [48,49,50,52,55,57]. Six studies explored the relationship between household size [48,49,50,51,55,57], and one study explored the relationship between location (rural/urban) and overweight and obesity [51]. The impact of parental perception of their child’s body weight on overweight or obesity was explored in two studies [56,57]. In all studies, overweight and obesity were defined using the World Health Organization growth standards [59] (Overweight: BMI ≥ 85 to ≤95 centiles, and obesity: BMI ≥ 95 centiles). The study characteristics and a summary of the findings are presented in Table 2 and Table 3.

**Table 2:** Characteristics and study quality of studies included in the review.

Study Author(s)	Year	Country	Study Design	Sample	Sample Age	Total Sample Size	Study Quality
Gewa [51]	2009	Kenya	CS ^1^	Children	3–5 years	1495	High
Okoye et al. [53]	2015	Nigeria	CS ^1^	Children	2–5 years	220	High
Sorrie et al. [48]	2017	Ethiopia	CS ^1^	Children	3–5 years	500	High
Wolde et al. [50]	2014	Ethiopia	CS ^1^	Children	3–5 years	358	High
Tadesse et al. [49]	2017	Ethiopia	CS ^1^	Children	3–6 years	462	High
Senbanjo et al. [54]	2007	Nigeria	CS ^1^	Children	0–5 years *	270	High
Said-Mohamed et al. [57]	2009	Cameroon	CS ^1^	Children	2–5 years	165	Medium
Kumordzie et al. [58]	2019	Ghana	CS ^1^	Children	4–6 years	889	High
Mamabolo et al. [55]	2005	South Africa	CS ^1^	Children	3 years	162	Medium
Klingberg et al. [56]	2020	South Africa	Qualitative study	Parents of preschoolers (3–5 years)	3–5 years	16	High
Wandia et al. [52]	2014	Kenya	CS ^1^	Children	3–6 years	320	Low

^1^ CS—Cross-sectional Study. * data for children with overweight/obesity was available for children aged 2–5 years old.

**Table 3:** Summary of findings of studies included in the review.

Study Author(s)	Home Environment Variables Studied (Exposure)	Exposure Measures/Tools of Measurement	Summary of Studies
Gewa et al. [51]	Maternal BMI	BMI (kg/m^2^)	Increase in maternal BMI is significantly associated with an increase in obesity/overweight among preschoolers (maternal overweight: OR = 1.83, 95% CI: 1.2, 2.81), maternal obesity: OR = 2.12, 95% CI (1.11, 4.07)).
	Maternal Education	Researcher-defined categories of Education level (No school/preschool, primary school, secondary school, post-secondary)	Increasing maternal education is significantly associated with an increase in obesity/overweight among preschoolers; maternal attainment of primary school (OR = 1.79, 95% CI: 1.09, 2.93) and secondary school (OR = 1.91, 95% CI: 1.05, 3.45). No significant effect of post-secondary levels of education (*p* > 0.05).
	Socioeconomic status: Household wealth index	Researcher-generated socioeconomic index scores based on Principal Component Analysis	No difference in household wealth index between children with obesity/overweight and non-obese/non-overweight children (*p* > 0.05)
	Household size	Survey data	Increasing household size is inversely associated with obesity/overweight among preschoolers. 7% reduction in odds of overweight/obesity with each additional person (*p* < 0.05)
	Household location	Survey data: rural versus urban living	A higher percentage of preschool children with overweight or obesity lived in urban areas compared to rural areas (21.83% (SE 2.83) versus 14.85% (SE1.15), *p* < 0.01)
Okoye et al. [53]	Daily consumption of sugar-sweetened beverages	Researcher-defined categories (>1 bottle in 2 days, ≤1 bottle in 2 days)	Increased consumption of sugar-sweetened beverages is significantly associated with an increase in obesity/overweight among preschoolers (crude OR = 18.98, 95% CI: 7.6, 47.40). Out of those children consuming > 1 bottle in 2 days, 88.46% had overweight/obesity (Chi^2^ = 55.34, *p* < 0.001).
	Daily total screen time	Researcher-defined categories (>1 h of TV, ≤1 h of TV)	No association between daily screen time (more than an hour or less) and obesity/overweight among preschoolers (*p* > 0.05).
	Food type	Researcher-defined categories (fruits/vegetables; grains, cereals, fried/fatty foods)	Increased consumption of fried/fatty foods is significantly associated with an increase in obesity/overweight among preschoolers (Crude OR = 2.16, 95% CI: 1.01, 4.61). Out of those children consuming more fatty foods, 28% were overweight/obese compared to 15.28% who were of normal weight (Chi^2^ = 3.97, *p* < 0.05).
Sorrie et al. [48]	Maternal education	Researcher-generated, Structured interviewer-administered questionnaire	Children of highly educated mothers were less likely to be overweight or obese. Children of mothers with secondary education were 65% less likely to be overweight and obese compared with children from mothers with no formal education (AOR = 0.35, 95% CI: 0.12, 0.96).
	Food consumption pattern	Food Frequency Questionnaire (FFQ)/past week	Increased consumption of sweet foods is significantly associated with overweight and obesity in preschoolers. Preschoolers who consumed sweet foods were 2.69 times more likely to be overweight/obese compared with children who did not consume sweet foods (AOR = 2.69, 95% CI: 1.21, 5.98).
	Dietary diversity	Dietary Diversity Score (DDS) (low, medium, high)/past 24 h	High dietary diversity is significantly associated with overweight/obesity among preschoolers. Preschool children with high dietary diversity scores were 3.73 times more likely to be overweight or obese compared with those with low dietary diversity scores (AOR = 3.73, 95% CI: 1.15, 12.54).
	Physical activity	WHO Global Physical Activity Questionnaire (GPAQ)/past week/month	Physical activity components of the GPAQ measured but results were not reported as a predictor of overweight/obesity in preschool children
	Daily total screen time	WHO Global Physical Activity Questionnaire (GPAQ)/past week/month	Watching TV > 2 h/day is significantly associated with an increase in overweight/obesity among preschoolers (AOR = 4.01, 95% CI: 2.22, 7.28).
	Socioeconomic status	Questionnaire; Possession or ownership of specified household assets	SES was not significantly associated with overweight or obesity among preschoolers (*p* > 0.05)
	Parental/caregiver employment	Questionnaire: Housewife, private, merchant, government employee, other	Variable measured, but no association reported in results
	Household size	Questionnaire	Variable measured, but no association reported in results
Wolde et al. [50]	Socioeconomic status	Researcher-generated, Structured interviewer-administered questionnaire; based on the possession of household assets	Preschool children with wealthier parents are approximately 3.5 times more likely to be overweight/obese than those with parents with low SES (AOR = 3.51, 95% CI: 1.30, 9.50).
	Food frequency consumption	Food Frequency Questionnaire (FFQ)/past month	Consumption of ice-cream (AOR = 3.84, 95% CI: 1.62, 7.09), sweet foods (AOR = 6.36, 95% CI: 1.88, 12.33) and fast foods (AOR = 8.69, 95% CI: 1.11, 13.50) is significantly associated with obesity/overweight compared with children who do not consume these foods.
	Dietary diversity	Dietary Diversity Score (DDS) (low, medium, high)/past 24 h	High dietary diversity is significantly associated with an increase in obesity/overweight among preschoolers (AOR = 3.48, 95% CI: 1.50, 8.10).
	Physical Activity Level	WHO Global Physical Activity Questionnaire (GPAQ)/past week/month	No significant association between total physical activity measures and obesity/overweight among preschoolers (*p* > 0.05)
	Maternal education	Researcher-generated, Structured interviewer-administered questionnaire	Variable measured, but no association reported in results
	Maternal employment	Researcher-generated, Structured interviewer-administered questionnaire	Variable measured, but no association reported in results
	Household size	Researcher-generated, Structured interviewer-administered questionnaire	Variable measured, but no association reported in results
Tadesse et al. [49]	Socioeconomic status: ownership of family car	Researcher-generated, Structured interviewer-administered questionnaire	Ownership of a family car is significantly associated with an increase in obesity/overweight among preschoolers (AOR = 3.43, 95% CI: 1.02, 11.49)
	Dietary diversity	Dietary Diversity Score (DDS) (poor, medium, high)	High dietary diversity is significantly associated with an increase in obesity/overweight among preschoolers (AOR = 5.12, 95% CI: 1.42, 18.47).
	Household size	Researcher-generated, Structured interviewer-administered questionnaire	Family size of less than five is associated with overweight and obesity in preschool children (AOR = 4.76, 95% CI: 1.84, 12.31).
	Maternal education	Researcher-generated, Structured interviewer-administered questionnaire	Variable measured, but no association reported in results
	Maternal employment	Researcher-generated, Structured interviewer-administered questionnaire	Variable measured, but no association reported in results
Senbanjo et al. [54]	Socioeconomic status	Researcher-generated, Structured interviewer-administered questionnaire	No significant association between socioeconomic status and obesity/overweight among 2–5-year-old children (*p* > 0.05)
Said-Mohamed et al. [57]	Socioeconomic status: Household economic index	Researcher-defined categories from PCA (low, middle and high)	No significant association between household economic index and overweight among preschoolers (*p* > 0.05)
	Food frequency consumption	Adapted Food Frequency Questionnaire (FFQ)/past month	No significant association between food frequency and overweight among preschoolers (*p* > 0.05)
	Perception of child’s height and weight	Researcher-defined categories; Weight grades (very thin, thin, average, fat, plump) and height grades (very short, short, average, tall, very tall)	Under-evaluation of child body weight by mothers is significantly associated with overweight among preschoolers (OR = 6.52, 95% CI: 2.34, 18.09). 79.5% of mothers of preschoolers with overweight underestimated their child’s weight
	Dietary diversity	Dietary Diversity Score (DDS)/past 24 h	No difference in dietary diversity between overweight and non-overweight children (*p* > 0.05)
	Maternal education	Interviewer-administered Questionnaire	Maternal education is not associated with overweight in preschool children (*p* > 0.05)
	Maternal employment	Interviewer-administered Questionnaire	No association of maternal employment status with childhood overweight (*p* > 0.05)
	Household size	Interviewer-administered Questionnaire	No significant differences in the number of household members between overweight and normal-weight preschoolers (*p* > 0.05)
	Maternal BMI	BMI (kg/m^2^)	No significant differences in maternal BMI between overweight and normal-weight children (*p* > 0.05)
Kumordzie et al. [58]	Dietary pattern	Food Frequency Questionnaire (FFQ)/past week	No significant association between snacking or cooked food dietary patterns and obesity/overweight among preschoolers (*p* > 0.05)
	Physical Activity	ActiGraph wGT3X-BT triaxial Accelerometry/1-week period	No significant association between physical activity and obesity/overweight among preschoolers when the model adjusted for sex since male children had higher levels of physical activity and lower percentage of body fat (*p* > 0.05)
	Maternal BMI	BMI (kg/m^2^)	An increase in maternal BMI is significantly associated with an increase in child fatness among preschoolers (standardised β = 0.10 (95% CI) (0.04, 0.16)
	Maternal Socioeconomic status	Open Data Kit (ODK) (version 1.4.7) software-administered questionnaire (open-source software downloadable from https://getodk.org/, accessed on 6 January 2022)	No significant direct association between maternal education or maternal household asset score and obesity/overweight among preschoolers (*p* > 0.05)
	Maternal education	Years spent in education	No significant direct association between maternal education and obesity/overweight among preschoolers (*p* > 0.05)
Mamabolo et al. [55]	Maternal occupational status	Researcher-developed, interviewer-administered structured questionnaire	Having a working mother was significantly associated with overweight among preschoolers (OR = 17.87, 95% CI: 8.24, 38.78)
	Housing structure	Researcher-developed, interviewer-administered structured questionnaire	The type of house of residence (traditional mud, brick or shack) was not significantly associated with overweight among preschoolers (*p* > 0.05)
	Household size	Researcher-developed, interviewer-administered structured questionnaire	No significant association between household size and overweight in preschool children (*p* > 0.05)
	Maternal education	Researcher-developed, interviewer-administered structured questionnaire	Maternal education was not significantly associated with overweight among preschoolers (*p* > 0.05)
Klingberg et al. [56]	Parental Perceptions of Childhood Obesity	Semi-structured in-depth interviews	Parents of preschoolers had varied perceptions of child body size and weight which were unrelated to health, e.g., comparisons and differences in appearances to peers or weight stigma. Three themes represented the perceptions of parents about their child’s weight, and these included “Growing Differently”, “The Right Way to Be”, “Weight is not Health”
Wandia et al. [52]	Socioeconomic status: Household possession of TV	Researcher-developed, interviewer-administered structured questionnaire	Possession of a TV in the household is associated with obesity in preschool children (Chi^2^ = 7.15, *p* = 0.006)
	Household ownership of a computer	Researcher-developed, interviewer-administered structured questionnaire	Possession of a computer in the household is associated with overweight (Chi^2^ = 3.95, *p* = 0.047) and obesity in preschool children (Chi^2^ = 7.12, *p* = 0.008)
	Mother’s education level	Primary, secondary, college or no education categories	Maternal education is significantly associated with obesity in preschool children (Chi^2^ = 20.4, *p* = 0.005). This factor was not entered into the logistical regression model by the authors
	Parental occupation status	Researcher-developed, interviewer-administered structured questionnaire	Obesity among preschoolers is associated with paternal occupation (Chi^2^ = 14.68, *p* = 0.002)

BMI—body mass index; FFQ—food frequency questionnaire; OR—odds ratio; CI—confidence interval; AOR—adjusted odds ratio; SES—socioeconomic status.

### 3.2. Quality Appraisal

No study was excluded based on the quality of evidence. However, studies with low quality were interpreted with caution. Eight studies were of high quality [48,49,50,51,53,54,56,58], two were of medium quality [55,57] and one was of low quality [52] (Table 3). In six of the eleven studies [48,50,51,52,55,57], the most frequent issues that compromised study quality were related to the non-disclosure or inadequate consideration of confounding. In the studies by Wandia et al. (2014), Okoye et al. (2015) and Senbanjo et al. (2007) [52,53,54], the exposure of interest was not measured using standard tools that have been validated for use in this age group across different geographical locations. Table 2 provides a summary of the quality of all studies that were included in this review.

### 3.3. Measures of the Home Environment and Overweight/Obesity among SSA Preschoolers

#### 3.3.1. Home Food Environment

Of the 11 included studies, 6 studies [48,49,50,53,57,58] with a total sample size of 2593 preschoolers measured child dietary intake within the home environment. Of these six studies, four [48,49,50,57] measured food intake using standard or adapted Food Frequency Questionnaires (FFQs) and dietary diversity scores (DDSs). The remaining two studies reported on child food consumption using unvalidated or novel methods of measurement, such as the evaluation of different dietary patterns by factor analysis [58] or researcher-defined classification of food types [53]. Four of the six studies that measured child food intake (comprising 1540 of the 2593 preschoolers) reported that the consumption of sugar-sweetened foods and beverages and increased dietary diversity are related to an increase in overweight and obesity among SSA preschoolers [48,49,50,53]. Two of the six cross-sectional studies [57,58] made up of 1053 preschoolers found no associations between dietary intake measured by food frequency, dietary diversity or dietary patterns and child BMI: (Table 3).

#### 3.3.2. Home Physical Activity Environment

Physical activity in the home environment was measured in 3 of the 11 studies included in this review [48,50,58]. When comprising 1747 preschoolers, physical activity was subjectively measured using the GPAQ in two studies [48,50] and objectively by accelerometry in one study [58]. No association was established between child physical activity or physical inactivity and obesity or overweight among SSA preschoolers [50,58]. Sorrie et al. [48] reported descriptive statistics for the GPAQ; however, these data were not used to explore the association with overweight/obesity: (Table 3).

#### 3.3.3. Home Media/Screen Time Environment

The home media environment was explored in three studies [48,52,53]. Daily screen time access was evaluated in two studies, with a combined sample size of 720 preschoolers [48,53]. Screen time was measured in the studies as >1 h/day [53] or >2 h/day [48]. In their study, Sorrie et al. [48] found that 3–5-year-old urban preschoolers in Ethiopia who spent more than 2 h a day watching television or playing games were four times more likely to be overweight or obese compared to those who spent less than 2 h. Conversely, Okoye et al. [53] reported that a minimum screen time of one hour per day was not associated with overweight/obesity among 2–5-year-old Nigerian preschoolers. An exposure of >2 h/day rather than an exposure of >1 h/day was shown to be associated with overweight and obesity among SSA preschoolers. Wandia [52] assessed the availability but not the duration of use of computers/TV in the home and reported an association between the ownership of a computer/TV with obesity among preschoolers: (Table 3).

### 3.4. Household Sociodemographic and Socioeconomic Factors

#### 3.4.1. Household Income and Wealth Status

There was no unifying and standard definition of household SES across all the selected studies. Of the 11 selected studies, 6 reported on compositely measured household wealth index or SES scores based solely on the possession of pre-specified household assets [48,50,51,54,57,58]. Compositely measured household wealth indices/asset scores were grouped as either tertiles [48,50,57] (e.g., low, middle/medium and high) or quintiles [51,54] (e.g., 1st, 2nd, 3rd, 4th and 5th). One study [58] measured household SES using asset scores without assigning them to any SES categories. One study by Wolde et al. [50] reported that children who were categorised in the high socioeconomic status tertiles were 3.5 times more likely to be overweight or obese compared to low socioeconomic status tertiles. There was no association between compositely measured SES (based on the possession of household assets) and overweight and obesity among SSA preschoolers in all other studies.

Although household assets were commonly evaluated as a composite measure in most studies, two studies reported a positive and significant association between the ownership of particular household assets, i.e., a family car [49] or a TV/computer [52] and excessive weight gain among SSA preschoolers: (Table 3).

#### 3.4.2. Maternal Body Mass Index

Three studies [51,57,58] examined the relationship between the BMI of mothers and overweight and obesity among a total of 2549 SSA preschoolers. All studies measured the BMI of mothers using the standard methodology of a height-corrected weight score (kg/m^2^). One study measured body fatness of children using the deuterium method [58], while the remaining two studies [51,57] measured overweight and obesity among preschoolers using standard BMI-for-age scores. Two studies [51,58] reported an association between maternal BMI and overweight and obesity among SSA preschoolers. Gewa et al. [51] report that maternal overweight and obesity are associated with increased odds of childhood overweight. Similarly, Kumordzie et al. [58] reported a significant association between maternal BMI and child percentage body fat. However, Said-Mohammed et al. [57] reported no significant difference in maternal BMI between children of normal weight and those overweight: (Table 3).

#### 3.4.3. Maternal Education

Eight studies investigated the association of the level of education of mothers/caregivers with overweight or obesity among SSA preschoolers [48,49,50,51,52,55,57,58]. Five studies [49,50,55,57,58] found no association or did not report any association between the level of formal education of mothers/caregivers and overweight and obesity among SSA preschoolers. For example, Mamabolo et al. [55] reported that a maternal education of either primary, secondary or higher (post-secondary education) was not associated with weight gain among 162 3-year-old rural South African preschoolers. In the Wolde et al. [50] and Tadesse et al. [49] studies, it was unclear whether maternal education was considered in the analysis since maternal education was measured but not discussed in the results. Although the majority of studies reported no association between maternal education and child overweight or obesity, three studies [48,51,52] did report an association, although the results are conflicted. Wandia et al. [52] reported that maternal education is a significant factor associated with child obesity but not overweight. However, despite this being identified as a significant predictor, this was not entered into the final logistic regression. This paper was deemed low quality, and the results should be interpreted with caution. The study by Sorrie et al. [48] demonstrated that the odds of having a child who was overweight or obese were reduced by 65% if mothers had attained secondary education. On the other hand, Gewa [51] showed that compared to mothers with no education, attaining primary or secondary education was positively associated with a 72% to 91% increase in the odds of having an overweight child: (Table 3).

#### 3.4.4. Occupational/Employment Status

Six studies of the 11 included studies in this review explored the occupation or employment status of mothers/caregivers [48,49,50,52,55,57]. Two of the six studies reported that preschool children of working parents [52,55] were more likely to be overweight or obese compared with children of non-working caregivers. In their study of 162 South African three-year-old children, Mamabolo et al. [55] reported that having a working mother increased the risk of being overweight by 17.87 times. Similarly, Wandia et al. [52] reported an association between paternal occupational status and obesity in preschool children; however, paternal occupation was not a significant predictor of obesity when entered into the logistic regression. This study was deemed low quality, and the results should be interpreted with caution. No relationship was reported for all other studies (Table 3) [48,49,50,57].

#### 3.4.5. Household Size

Six studies measured household size [48,49,50,51,55,57]. Two of these studies measured this variable but did not report its association with overweight and/or obesity in preschool children [48,50]. Two out of six studies [49,51] demonstrated that larger household family sizes reduced the risk of overweight and obesity in preschool children. Tadesse et al. [49], in their study of Ethiopian preschool children, reported that children living in a household of less than five were more likely to be overweight or obese compared to those living in a family containing five or more members. Similarly, Gewa [51] reported that Kenyan preschoolers living in large household sizes had lower odds of overweight and obesity. A 7% reduction in odds of being overweight or obese was reported for each additional household member. Mamabolo et al. [55] and Said-Mohammed et al. [57] reported no significant differences in the number of household members between overweight and normal-weight preschoolers: (Table 3).

#### 3.4.6. Location: Urban Versus Rural Living

One study explored the relationship between location (rural/urban) and overweight and obesity [51]. Gewa [51] reported that a significantly higher percentage of Kenyan preschool children with overweight and obesity lived in urban areas, whilst a higher percentage of children who were not overweight or obese lived in rural areas: (Table 3).

#### 3.4.7. Parental Perception of Child Body Weight

Parental perception of a child’s body weight was identified as a relevant social aspect of the home environment in two studies [56,57]. Evidence for this aspect of the home environment was demonstrated in one qualitative study of the perceptions and attitudes of parents of SSA preschoolers to overweight and obesity [56]. In this study, caregivers often had varied perceptions of the body size and weight of their children, which were unrelated to health. Examples of these perceptions included comparisons and differences in appearances to their peers or the potential cultural stigma attached to weight gain only when it was considered excessive. Similarly, the quantitative study by Said-Mohamed et al. [57] reported that mothers who underestimated their child’s weight were more likely to have a child with overweight or obesity.

## 4. Discussion

The aim of the current review was to explore which aspects of the modified obesogenic home environment model are associated with overweight and obesity in preschool children in Sub-Saharan preschool children. Eleven studies from six Sub-Saharan countries were identified, including ten quantitative papers and one qualitative paper. Overall, the results demonstrate that the home food environment, through the types of foods offered to preschoolers, and sociodemographic factors, through maternal BMI, are key aspects contributing to overweight and obesity among SSA preschoolers. The media home environment, physical activity and household socioeconomic status (income, education and employment) were not identified as factors associated with preschool overweight and obesity in the current review.

### 4.1. Home Food Environment

The evidence from this review suggests that dietary intake plays an important role in the development and maintenance of overweight and obesity in preschool children. In the current review, four out of six studies reported an association between dietary intake and overweight and obesity among SSA preschoolers [48,49,50,53]. Specifically, these studies found that an increased intake of fried/fatty foods [53], fast foods [50], sweet foods [48,50] and sugar-sweetened drinks/beverages [53] were associated with overweight and obesity among the population of 2–6-year-old preschoolers. These findings are consistent with those reported in other studies [60,61,62] that have shown that the consumption of energy-dense and nutrient-poor foods is associated with weight gain in children. In our review, Mezie-Okoye et al. [53] reported an association between the consumption of sugar-sweetened beverages and an increased likelihood of overweight or obesity in 2–5-year-old Nigerian urban preschoolers. Sorrie et al. [48] also found that those who were frequently offered sweet, high-energy dense foods at home were 2–3 times more likely to be overweight/obese compared to those who were not offered these foods. Wolde et al. [50] also demonstrated that 3–5-year-old urban Ethiopian children who were offered ice cream and sweet foods at home were 4 to 7 times more likely to be overweight and obese, respectively, compared to those who did not. Those who were frequently offered fast foods were also 8–9 times more likely to develop overweight and obesity compared to those who were not.

Contrary to evidence of an inverse relationship between dietary diversity and excessive weight gain in many “western” or developed countries [63], our review showed that increased dietary diversity was associated with overweight and obesity in children [37,38,39]. Specifically, Sorrie et al. [48] demonstrated that the likelihood of becoming overweight and obese was 3–4 times higher for 3–5-year-old urban Ethiopian preschoolers with a high dietary diversity score than those with a low dietary diversity score. Wolde et al. [50] also showed that children with a high dietary diversity score were 3–4 times more likely to be overweight or obese. This finding was also confirmed in the Tadesse et al. [49] study, which also demonstrated that 3–6-year-old Ethiopian preschoolers with a high dietary diversity were five times more likely to be overweight or obese. This unexpected finding may reflect overconsumption of certain food groups over other food groups, such as the consumption of more processed high-energy dense, nutrient-poor foods over fruits and vegetables. There are also suggestions that even though dietary diversity scores may be high among some child populations, the overconsumption of certain foods over others may reflect cultural or seasonal dietary patterns [64]. This finding may also be the result of the consumption of large portion sizes of different food groups, a feature that the dietary diversity score assessment from these studies may not have captured, as suggested by Sorrie et al. (2017) [48].

Despite the majority of studies demonstrating an association between food consumption and overweight and obesity, two studies did not show this association [57,58]. In their study, Kumordzie et al. [58] reported that a dietary snacking pattern or one consisting mainly of home-cooked foods was not important in contributing to overweight/obesity among Ghanaian preschoolers aged 4–6 years. Said-Mohamed et al. [57] also found that dietary diversity scores for overweight and non-overweight 2–4-year-old Cameroonian preschoolers were similar. One possible reason for this inconsistency could be the cross-sectional nature of the studies identified. Obesity develops over time [65], and significant weight gain over a protracted period of time may not always be detectable in a single snapshot of time. Moreover, for these studies, considerable variations exist in the measurement tools and analysis of dietary intake. For example, the study by Kumordzie et al. [58] stated that their dietary data collection tool was not designed specifically to capture the energy density of meals served to children.

#### 4.1.1. Home Physical Activity Environment

This review identified three studies that examined the role of the home physical activity environment [48,50,58]. Among these studies, Wolde et al. [50] measured the physical activity levels (classified as either low, moderate or high) of 3–5-year-old preschoolers in urban Ethiopia using the Global Physical Activity Questionnaire (GPAQ) and found no association between physical activity and overweight and obesity. Kumordzie et al. [58] examined the physical activity of 4–6-year-old urban preschoolers in Ghana using accelerometers and found that vector magnitude counts, which represent the intensity of body movements, were not associated with adiposity when accounting for age and sex.

It was suggested in a recent review of the home environment and adiposity in children that inconsistencies in the relationship between physical activity and child BMI are to be expected, in part because of weaknesses in the tools that were used for the measurement of physical activity in children [18]. However, despite the identified issues, further work is required in the SSA context to further explore the relationship between physical activity and body weight in young children. Sedentary behaviour can also be due to a lack of access to outdoor spaces [66,67]. This was not explored in any of the identified papers in the current review and would be an interesting area for future research, particularly in urban-dwelling children with limited access to outdoor spaces. Further, the interaction between the home media environment and physical activity in SSA children is unclear.

#### 4.1.2. Home Media Environment

Only three studies were identified in the current review that explored the role of the home media environment and excess weight in preschool children, though with inconclusive findings [48,52,53]. Sorrie et al. showed that a minimum screen time of two hours per day was associated with an increased risk of overweight/obesity in 3–5-year-old urban Ethiopian preschoolers [48]. Conversely, Okoye et al. [53] reported no association between minimum screen time of one hour per day and risk of overweight/obesity among 2–5-year-old Nigerian preschoolers. When the availability of media devices such as television and computers in the home environment was explored, an association with obesity was observed in Kenyan preschool children aged 3–6 years old [52]. However, in that study, only the access was measured and not the duration of exposure. It is likely that the overall results are unclear due to the limited number of studies identified that focused specifically on the home media environment. These current findings contrast with the recent systematic review by Kininmonth et al. [18], who reported that the home media environment, specifically the access to electronic devices, was most consistently associated with excess adiposity in children. Despite these inconsistencies, the home media environment is an important area for future investigations in SSA if technology continues to diversify and children start to gain access to media devices in the home environment.

### 4.2. Sociodemographic and Socioeconomic Factors

#### 4.2.1. Maternal BMI

Evidence for the relationship between maternal BMI and childhood obesity reported in two studies with the largest sample sizes showed a positive association [51,58], consistent with findings from other studies [68,69]. Evidence from the Kenyan Demographic and Health Survey of children aged 3–5 years [51] found that children who were overweight or obese were approximately twice as likely to have mothers who were also overweight or obese compared with children who had normal weight. Similarly, Kumordzie et al. [58] reported a positive relationship between maternal and child BMI in Ghanaian 4–6-year-olds, adjusting for age and sex. The relationship between maternal BMI and child overweight/obesity could result from the influence of non-genetic factors such as parental modeling of feeding behaviour [70]. Behaviour is learned, and it is possible that obese and overweight mothers exhibit obesogenic dietary patterns that children learn [70]. Mothers who are overweight or obese may be more likely to consume high energy-dense and less nutrient-dense foods themselves, resulting in these foods being more available and accessible to children [12,71,72,73,74]. Obese mothers may also exert their influence on obesity and feeding behaviour in children through feeding practices [70,75] such as restriction, pressure to eat [76] or using food as a reward [77]. Since mothers are often the nutritional gatekeepers [27], more research is needed to explore the relationship between parental body weight, dietary provision, parental feeding practices and styles, and child body weight in the SSA context. Contrary to these findings, one study included in the review by Said-Mohamed et al. [57] reported that mothers of overweight and non-overweight children did not differ in terms of BMI. However, the sample sizes in this study are most likely too small to detect any real differences between the study groups (*n* = 36 for mothers of children with overweight and *n* = 37 for mothers of non-overweight children).

#### 4.2.2. Household Income and Wealth Status

The evidence for the relationship between household income or wealth status and excess weight among SSA preschoolers remains unclear. Wolde et al. [50] demonstrated that 3–5-year-old Ethiopian preschoolers in high SES households were 3–4 times more likely to be overweight or obese, which is similar to findings from other studies that have shown that higher-income households in developing countries are more likely to have children who are overweight or obese [21]. Similarly, Tadesse et al. [49] and Wandia et al. [52] reported that possession of a car and a tv/computer, respectively, are associated with overweight and obesity in preschool children. However, the remaining included studies which included some measure of income or wealth reported no association, suggesting that the income and wealth status of households may not be reliable predictors of obesity and overweight among preschool children in Sub-Saharan Africa [48,51,54,57,58]. This finding must, however, be interpreted with caution since potential issues with the assessment/measurement of wealth may account for this discrepancy. It has been suggested that the choice of household assets and analyses methods for evaluating household wealth varies between studies and could potentially obscure the wealth status of a household [78].

#### 4.2.3. Maternal Education

Evidence suggests that excessive weight gain in children is associated with lower levels of maternal education, as shown in studies conducted in Western countries [79,80], with the assumption that this mediates the relationship between nutrition knowledge, the mother’s perception of the child’s body weight, maternal feeding styles [81], maternal feeding practices [82] and maternal modelling of nutrition and physical activity behaviour. However, this association was not evidenced in our review. One study [51] reported that higher maternal education is associated with increased odds of preschoolers being overweight, while another study [48] demonstrated the opposite effect, i.e., an increased odds of overweight and obesity among SSA preschoolers with decreasing levels of education among their mothers/caregivers. The remaining six studies [49,50,52,55,57,58] either did not clearly report the results [49,50,52] or reported no notable relationships between the level of formal education of mothers/caregivers and overweight and obesity among SSA preschoolers [55,57,58]. These inconsistencies might be due to different study designs and inconsistent reporting of results. Alternatively, these inconsistencies might suggest that the distribution of maternal education levels may be similar across obese and non-obese groups or that maternal education might not be a strong predictor of overweight or obesity among SSA preschoolers.

#### 4.2.4. Occupational/Employment Status

Most studies [48,49,50,57] included in this review did not report any relationship between caregiver occupational status and overweight and obesity in preschool children. It is important to note that three studies examined caregiver occupational or employment status but did not explicitly report the association with overweight or obesity in the Section 3 [37,38,39]. However, two studies reported a relationship between parental occupational status and child overweight and obesity [52,55]. The findings from the current review support the recent work of Oddo et al. [83], who reported no association between maternal employment and overweight in preschool children in low- and middle-income countries when data across 45 LMICs were pooled. However, Oddo et al. [83] reported several interesting findings specific to SSA. For example, increased odds of overweight were associated with formal maternal employment in Ghana and Kenya and increased odds of being overweight were associated with informal maternal employment in Cameroon and Chad. It is unclear why these inconsistencies occur. The general low prevalence of overweight and obesity in some areas compared to others may be a factor. Oddo et al. reported that maternal education moderated the association between employment status and childhood overweight and obesity [83]. Children of formally employed mothers with higher education had higher odds of being overweight compared to non-employed mothers who were highly educated. Mixed results regarding this association are also observed in high-income countries, with some studies from Europe reporting limited evidence [84] whilst others from the UK and USA report a strong relationship [85,86].

The relationship between child weight, maternal/caregiver occupational status and maternal education is complex and appears to be further related to other factors such as socioeconomic status, cultural norms and values, access and availability of particular foods. Future research would benefit from comprehensive consideration of related factors, some of which were discussed in but not limited to those in the current review. For example, access to childcare, type of work carried out, working hours and the number of jobs parents have.

#### 4.2.5. Household Size

Our findings with regard to the association between household/family size and obesity in preschoolers were mixed with evidence reporting no association [55,57] and evidence supporting a protective effect of larger families on the risk of overweight and obesity [49,51]. The relationship between family size and risk of overweight/obesity was previously reported in a 2014 review by Keino et al. [87]. They suggested that increasing numbers per household were associated with a reduced prevalence of overweight in young children and adolescents due to increased competition for available food. A USA-based study [88] reported similar findings. Eating together as a family at home reduced maternal work, and increased adult supervision of children are potential mechanisms through which family size may be protective of childhood obesity [88]. Understanding how family size may impact child overweight/obesity, specifically in SSA, is warranted.

#### 4.2.6. Location: Urban Versus Rural Living

Evidence to support the association between location (urban versus rural) and overweight and obesity in urban preschool children were supported in our review [51]. Rapid urbanisation has previously been identified as a key driver of overweight and obesity [89,90]. Steyn et al. reported the prevalence of childhood overweight using the South African National Food Consumption Survey; these data demonstrate that the highest prevalence of overweight was observed in urban children and the lowest prevalence of overweight in rural children living on farms [28]. However, a recent study by Madisse and Letamo (2017) examined the prevalence of overweight in SSA females and reported that rural African women were at increased or higher risk of being overweight or obese compared to urban-dwelling women [91]. The authors noted that the simple dichotomy of urban/rural living is insufficient to understand overweight in SSA and that the relation between weight status and place of residence is highly complex, with many studies failing to take into account household wealth [91].

#### 4.2.7. Parental Perception of Child Body Weight

Qualitative evidence from this review suggests that culturally motivated perspectives of body weight in Sub-Saharan Africa may be critical in influencing the food choices and feeding practices of SSA parents and caregivers [92]. Parents of preschoolers had varied perceptions of their child’s body size and body weight, which were unrelated to health [56]. Excess weight was rarely reported as a concern, and parents rarely reported an association between what their children ate and their child’s body weight. Excess weight was only seen as a concern if it resulted in child immobility and the development of chronic diseases such as diabetes. Interestingly, despite the positive connotations of excess weight being identified, the link between obesity stigma and bullying was discussed by some participants: highlighting the key role played by parents in the home environment and how they might influence body weight. Similarly, the current review provided evidence that mothers who underestimate their child’s weight were more likely to have a child with excess weight [57]. Very little is known about parental feeding practices and feeding styles in the SSA context and how different social and cultural norms regarding eating behaviours might impact the development of excess weight in young children.

### 4.3. Limitation of Study and Recommendations

Our review followed a rigorous approach, adhering to standard practices for accessing and reporting research; however, there are some limitations that need to be acknowledged. The studies identified represented data from 6 out of approximately 46 Sub-Saharan African countries. Similarly, regional differences in the components of the modified home environment model were not explored in the current review and should be considered in future investigations. All the studies that were identified were of a cross-sectional nature, limiting the interpretation of the effect of home environment factors on child behaviour and weight gain to a single point in time. Our review also identified only one qualitative study, limiting our understanding of the depth of the relationship between the home environment and childhood overweight and obesity. Finally, wide variations existed in the measurement of key exposure variables such as food intake/dietary intake or physical activity, which limited the aggregation of the data into a meta-analysis.

## 5. Conclusions

Overall, the results of the current review highlight the paucity of studies exploring factors in the home environment associated with overweight and obesity in preschool children in Sub-Saharan Africa. Important key contributors to overweight and obesity identified in the current review include the home food environment and maternal BMI. However, the evidence for all other factors explored is mixed and remains unclear. It is important that further work is conducted in this under-researched area using standardised, validated measures of important aspects such as food intake, physical activity, parental feeding practices and styles. Longitudinal work would also help establish associations over time. Until a strong evidence base is established, effective interventions cannot be developed, thus highlighting the need for this critical work to be undertaken.

## Figures and Tables

**Table 1:** Inclusion and exclusion criteria for studies that were included in the review.

Component of Research Question	Inclusion Criteria	Exclusion Criteria
Population	Studies focused on preschool children aged 2–6 years of age, attending nursery or kindergarten schools, or their parents/caregivers. Studies where age-specific data could be extracted.	Studies conducted among infants and children aged below 2 years and those above 6 years Studies that included children with diagnosed eating/feeding disorders or long-term illnesses Child age not reported
Exposure	Included any measurements of components of the home environment (e.g., socioeconomic status, food security, dietary diversity, parent feeding styles/practices)	Studies conducted in healthcare or school settings
Context	Conducted in a Sub-Saharan African country	Studies conducted outside the Sub-Saharan Africa region, for example, those including African immigrants resident in Europe
Outcome	Reported on overweight and obesity among preschoolers	Nutritional status, overweight or obesity was studied as a predictor of other outcomes such as infections, cognitive behaviour or illness Nutritional status was measured based on the daily consumption of micronutrients Studies that measured and reported solely on other forms of nutritional status such as “underweight”, “wasting”, “stunting” or individual-level “stunting with obesity” Studies that measured overweight and obesity as an outcome among Sub-Saharan preschoolers but did not measure any associations with any component of the home environment, e.g., prevalence studies of obesity and overweight among Sub-Saharan preschoolers
Study design	Cross-sectional or cohort studies	Case studies, case series, case-control studies or trials Systematic or Narrative Reviews Grey literature Non-English language publications Conference abstracts/paper with no full text

## Data Availability

Not applicable.

## Supplementary Materials

The following supporting information can be downloaded at: https://www.mdpi.com/article/10.3390/nu14091706/s1, Search terms for all databases.
