# Supplementary material for: Risk Factors for Overweight and Obesity within the Home Environment of Preschool Children in Sub-Saharan Africa: A Systematic Review
Source: Nutrients. 2022 Apr 20;14(9):1706. doi: 10.3390/nu14091706 (PMC9100775; doi:10.3390/nu14091706)
Supplement: Supplementary file 1 [file nutrients-14-01706-s001.zip › nutrients-1616579-supplementary.pdf]

Supplementary Materials: Literature Search strategy and records retrieved from each database

MEDLINE: [*n* = 765]

#	Searches	Results
1	Risk Factors/	914213
2	Association/	4155
3	Child, Preschool/	971519
4	Schools, Nursery/	1497
5	exp "Africa South of the Sahara"/	236892
6	Pediatric Obesity/	11842
7	Nutritional Status/	50583
8	Overnutrition/ or Hyperphagia/	3983
9	Obesity/	200098
10	Overweight/	29330
11	Adiposity/	14523
12	body mass index/	141707
13	Body Weight/	196020
14	(risk factor* or predispo* factor* or determin* or predict* or relat* or associat* or correlat*).mp. [mp=title, abstract, original title, name of substance word, subject heading word, floating sub-heading word, keyword heading word, organism supplementary concept word, protocol supplementary concept word, rare disease supplementary concept word, unique identifier, synonyms]	13987945
15	(preschool* or pre school* or pre-school* or toddler* or kindergarten* or nursery school* or day care center* or day care centre* or daycare center* or daycare centre* or day-care center* or day-care centre* or pre-primary school* or pre primary school* or creche*).mp. [mp=title, abstract, original title, name of substance word, subject heading word, floating sub-heading word, keyword heading word, organism supplementary concept word, protocol supplementary concept word, rare disease supplementary concept word, unique identifier, synonyms]	989973
16	(Sub-sahara* Africa or SSA or east Africa or west Africa or central Africa or southern Africa or Angola or Benin or Botswana or Burkina Faso or Burundi or Cameroon or Cape Verde or Central African Republic or Chad or Comoros or Congo or Democratic Republic Congo or Cote d'Ivoire or Ivory Coast or Djibouti or Equatorial Guinea or Eritrea or Ethiopia or Gabon or Gambia or Ghana or	502052

	Guinea or Guinea-Bissau or Kenya or Lesotho or Liberia or Madagascar or Malawi or Mali or Mauritania or Mauritius or Mozambique or Namibia or Niger or Nigeria or Rwanda or Sao Tome or Senegal or Seychelles or Sierra Leone or Somalia or South Africa or Sudan or Swaziland or Eswatini or Tanzania or Togo or Uganda or Zambia or Zimbabwe).mp. [mp=title, abstract, original title, name of substance word, subject heading word, floating sub-heading word, keyword heading word, organism supplementary concept word, protocol supplementary concept word, rare disease supplementary concept word, unique identifier, synonyms]	
17	(nutritional status or obes* or overweight or overnutrition or over nutrition or overeat* or overfeed* or adipos* or fatness or bmi z score* or body weight*).mp. [mp=title, abstract, original title, name of substance word, subject heading word, floating sub-heading word, keyword heading word, organism supplementary concept word, protocol supplementary concept word, rare disease supplementary concept word, unique identifier, synonyms]	892551
18	(home* or house* or household* or household-level or mother* or maternal or father* or paternal or parent* or family$ or caregiver*).mp. [mp=title, abstract, original title, name of substance word, subject heading word, floating sub-heading word, keyword heading word, organism supplementary concept word, protocol supplementary concept word, rare disease supplementary concept word, unique identifier, synonyms]	2690966
19	(home* or house* or household-level or mother* or maternal or father* or paternal or parent* or family$ or caregiver*).af.	2970051
20	1 or 2 or 14	13987945
21	3 or 4 or 15	990107
22	5 or 16	507859
23	6 or 7 or 8 or 9 or 10 or 11 or 12 or 13 or 17	943609
24	18 and 20 and 21 and 22 and 23	906
25	19 and 20 and 21 and 22 and 23	954
26	limit 25 to (english language and yr="2000 -2021")	765

EMBASE: [*n* = 733]

#	Searches	Results
1	risk factor/	1193752
2	association/	40255
3	predictor variable/	29034
4	disease predisposition/	97663

5	preschool child/	586251
6	nursery school/	1031
7	toddler/	5718
8	kindergarten/	3262
9	day care/	12522
10	exp "Africa south of the Sahara"/	278128
11	childhood obesity/	18127
12	nutritional status/	73428
13	overnutrition/	6047
14	hyperphagia/	6510
15	obesity/	476454
16	body mass/	529973
17	body weight/	333931
18	(risk factor* or susceptib* or prone* or predispo* factor* or determin* or predict* or relat* or associat* or correlat*).mp. [mp=title, abstract, original title, name of substance word, subject heading word, floating sub-heading word, keyword heading word, organism supplementary concept word, protocol supplementary concept word, rare disease supplementary concept word, unique identifier, synonyms]	17728342
19	(preschool* or pre school* or pre-school* or toddler* or kindergarten* or nursery school* or day care center* or day care centre* or daycare center* or daycare centre* or day-care center* or day-care centre* or pre-primary school* or pre primary school* or creche*).mp. [mp=title, abstract, original title, name of substance word, subject heading word, floating sub-heading word, keyword heading word, organism supplementary concept word, protocol supplementary concept word, rare disease supplementary concept word, unique identifier, synonyms]	617208
20	(Sub-sahara* Africa or SSA or east Africa or west Africa or central Africa or southern Africa or Angola or Benin or Botswana or Burkina Faso or Burundi or Cameroon or Cape Verde or Central African Republic or Chad or Comoros or Congo or Democratic Republic Congo or Cote d'Ivoire or Ivory Coast or Djibouti or Equatorial Guinea or Eritrea or Ethiopia or Gabon or Gambia or Ghana or Guinea or Guinea-Bissau or Kenya or Lesotho or Liberia or Madagascar or Malawi or Mali or Mauritania or Mauritius or Mozambique or Namibia or Niger or Nigeria or Rwanda or Sao Tome or Senegal or Seychelles or Sierra Leone or Somalia or South Africa or Sudan or Swaziland or Eswatini or Tanzania or Togo or Uganda or Zambia or Zimbabwe).mp. [mp=title, abstract, original title, name of substance word, subject heading word, floating sub-heading word, keyword heading	520713

	word, organism supplementary concept word, protocol supplementary concept word, rare disease supplementary concept word, unique identifier, synonyms]	
21	(nutritional status or obes* or overweight or overnutrition or over nutrition or overeat* or overfeed* or adipos* or fatness or bmi z score* or body weight*).mp. [mp=title, abstract, original title, name of substance word, subject heading word, floating sub-heading word, keyword heading word, organism supplementary concept word, protocol supplementary concept word, rare disease supplementary concept word, unique identifier, synonyms]	1293712
22	(home* or house* or household* or household-level or mother* or maternal or father* or paternal or parent* or family$ or caregiver*).mp. [mp=title, abstract, original title, name of substance word, subject heading word, floating sub-heading word, keyword heading word, organism supplementary concept word, protocol supplementary concept word, rare disease supplementary concept word, unique identifier, synonyms]	3383483
23	(home* or house* or household* or household-level or mother* or maternal or father* or paternal or parent* or family$ or caregiver*).af.	3962634
24	1 or 2 or 3 or 4 or 18	17744942
25	5 or 6 or 7 or 8 or 9 or 19	624365
26	10 or 20	523175
27	11 or 12 or 13 or 14 or 15 or 16 or 17 or 21	1572761
28	23 and 24 and 25 and 26 and 27	849
29	limit 28 to (english language and yr="2000 - 2021")	733

CINAHL: [*n* = 212]

#	QUERY	RESULTS
S1	(MH "Risk Factors+")	223,487
S2	risk factors or contributing factors or predisposing factors or predictor or cause or influencing factors or determinants or relationship or correlation	1,458,698
S3	S1 OR S2	1,471,329
S4	(MH "Child, Preschool") OR (MH "Schools, Nursery")	222,756
S5	(MH "Child Day Care")	2,793
S6	preschool or kindergarten or early childhood education or nursery school or toddlers or day care center or day care centre or pre-primary school or creche	233,726
S7	S4 OR S5 OR S6	234,188
S8	(MH "Africa South of the Sahara+") OR (MH "Africa, Western") OR (MH "Africa, Southern") OR (MH "Africa, Northern") OR (MH "Africa, Eastern") OR (MH "Africa, Central")	78,033

S9	Sub-Saharan Africa or SSA or east Africa or west Africa or central Africa or southern Africa or Angola or Benin or Botswana or Burkina Faso or Burundi or Cameroon or Cape Verde or Central African Republic or Chad or Comoros or Congo or Democratic Republic Congo or Cote d'Ivoire or Ivory Coast or Djibouti or Equatorial Guinea or Eritrea or Ethiopia or Gabon or Gambia or Ghana or Guinea or Guinea-Bissau or Kenya or Lesotho or Liberia or Madagascar or Malawi or Mali or Mauritania or Mauritius or Mozambique or Namibia or Niger or Nigeria or Rwanda or Sao Tome or Senegal or Seychelles or Sierra Leone or Somalia or South Africa or Sudan or Swaziland or Eswatini or Tanzania or Togo or Uganda or Zambia or Zimbabwe	106,956
S10	S8 OR S9	107,818
S11	(MH "Pediatric Obesity")	16,178
S12	(MH "Nutritional Status")	17,085
S13	(MH "Hyperphagia")	1,003
S14	(MH "Obesity+")	109,272
S15	(MH "Body Mass Index")	90,757
S16	(MH "Body Weight+")	166,183
S17	childhood obesity or obese children or overweight children or nutritional status or fatness or adiposity or overnutrition or overeat or overfeed or bmi z score or bmi or body weight or	219,507
S18	S11 OR S12 OR S13 OR S14 OR S15 OR S16 OR S17	301,563
S19	(MH "Home Environment")	11,784
S20	home environment or family influence or family environment or household or caregiver or mother or father or paternal or maternal or parent	424,177
S21	S19 OR S20	424,177
S22	S3 AND S7 AND S10 AND S18 AND S21	258
S23	S3 AND S7 AND S10 AND S18 AND S21 (Limiters - Published Date: 20000101-20211231; English Language; Research Article Expanders - Apply equivalent subjects Search modes - Boolean/Phrase)	212

<u>SCOPUS: [*n* = 318]</u>

SEARCH TERMS	RESULTS
TITLE-ABS (preschool* OR "pre school*" OR pre-school* OR kindergarten* OR "nursery school*" OR "day care center*" OR "day care centre*" OR "daycare center*" OR "daycare centre*" OR "day-care center*" OR "day-care centre*" OR "pre-primary school*" OR "pre primary school*" OR creche*) AND ("nutritional status" OR obes* OR overweight OR overnutrition OR "over nutrition" OR overeat* OR overfeed* OR adipos* OR fatness OR "bmi z-score*" OR "body weight*") AND ("Sub-sahara* Africa" OR ssa OR "east Africa" OR "West Africa" OR "Central Africa" OR "southern Africa" OR angola OR benin OR botswana OR burkina AND faso OR burundi OR cameroon OR "Cape Verde" OR "Central African Republic" OR chad OR comoros OR congo OR "Democratic Republic Congo" OR "Cote d'Ivoire" OR "Ivory Coast" OR djibouti OR "Equatorial Guinea" OR eritrea OR ethiopia OR gabon OR gambia OR ghana OR guinea OR guinea-bissau OR kenya OR lesotho OR liberia OR madagascar OR malawi OR mali OR mauritania OR mauritius OR mozambique OR namibia OR niger OR nigeria OR rwanda OR " Sao Tome" OR senegal OR seychelles OR "Sierra Leone" OR somalia OR "South	433 document results

Africa" OR sudan OR swaziland OR eswatini OR tanzania OR togo OR uganda OR zambia OR zimbabwe)	
TITLE-ABS (preschool* OR "pre school*" OR pre-school* OR kindergarten* OR "nursery school*" OR "day care center*" OR "day care centre*" OR "daycare center*" OR "daycare centre*" OR "day-care center*" OR "day-care centre*" OR "pre-primary school*" OR "pre primary school*" OR creche*) AND ("nutritional status" OR obes* OR overweight OR overnutrition OR "over nutrition" OR overeat* OR overfeed* OR adipos* OR fatness OR "bmi z-score*" OR "body weight*") AND ("Sub-sahara* Africa" OR ssa OR "east Africa" OR "West Africa" OR "Central Africa" OR "southern Africa" OR angola OR benin OR botswana OR burkina AND faso OR burundi OR cameroon OR "Cape Verde" OR "Central African Republic" OR chad OR comoros OR congo OR "Democratic Republic Congo" OR "Cote d'Ivoire" OR "Ivory Coast" OR djibouti OR "Equatorial Guinea" OR eritrea OR ethiopia OR gabon OR gambia OR ghana OR guinea OR guinea-bissau OR kenya OR lesotho OR liberia OR madagascar OR malawi OR mali OR mauritania OR mauritius OR mozambique OR namibia OR niger OR nigeria OR rwanda OR "Sao Tome" OR senegal OR seychelles OR "Sierra Leone" OR somalia OR "South Africa" OR sudan OR swaziland OR eswatini OR tanzania OR togo OR uganda OR zambia OR zimbabwe) AND PUBYEAR > 2000 AND PUBYEAR < 2021 AND (LIMIT-TO (LANGUAGE , "English"))	318 document results

WEB OF SCIENCE:[*n* = 117]

#	SEARCH RESULTS	SEARCH QUERY
# 1	41,928	(TI=(preschool* or "pre school*" or pre-school* or kindergarten* or "nursery school*" or "day care center*" or "day care centre*" or "daycare center*" or "daycare centre*" or "day-care center*" or "day-care centre*" or "pre-primary school*" or "pre primary school*" or creche*))
# 2	55,496	(AB=(preschool* or "pre school*" or pre-school* or kindergarten* or "nursery school*" or "day care center*" or "day care centre*" or "daycare center*" or "daycare centre*" or "day-care center*" or "day-care centre*" or "pre-primary school*" or "pre primary school*" or creche*))
# 3	74,706	#2 OR #1
# 4	283,009	(TI=("nutritional status" or obes* or overweight or overnutrition or "over nutrition" or overeat* or overfeed* or adipos* or fatness or "bmi z-score*" or "body weight*"))
# 5	575,025	(AB=("nutritional status" or obes* or overweight or overnutrition or "over nutrition" or overeat* or overfeed* or adipos* or fatness or "bmi z-score*" or "body weight*"))
# 6	705,972	#5 OR #4
# 7	480,653	(TI=("Sub-sahara* Africa" or SSA or "east Africa" or "West Africa" or "Central Africa" or "southern Africa" or Angola or Benin or Botswana or Burkina Faso or Burundi or Cameroon or "Cape Verde" or "Central African Republic" or Chad or Comoros or Congo or "Democratic Republic Congo" or "Cote d'Ivoire" or "Ivory Coast" or Djibouti or "Equatorial Guinea" or Eritrea or Ethiopia or Gabon or Gambia or Ghana or Guinea or Guinea-Bissau or Kenya or Lesotho or Liberia or Madagascar or Malawi or Mali or Mauritania or Mauritius or Mozambique or Namibia or Niger or Nigeria or Rwanda or "Sao Tome" or Senegal or Seychelles or "Sierra Leone" or Somalia or "South Africa" or Sudan or Swaziland or Eswatini or Tanzania or Togo or Uganda or Zambia or Zimbabwe))
# 8	467,119	(AB=("Sub-sahara* Africa" or SSA or "east Africa" or "West Africa" or "Central Africa" or "southern Africa" or Angola or Benin or Botswana or Burkina Faso or Burundi or Cameroon or "Cape Verde" or "Central African Republic" or Chad or Comoros or Congo or "Democratic Republic Congo" or "Cote d'Ivoire" or "Ivory Coast" or Djibouti or "Equatorial Guinea" or Eritrea or Ethiopia or Gabon or Gambia or Ghana or Guinea or Guinea-Bissau or Kenya or Lesotho or Liberia or Madagascar or Malawi or Mali or Mauritania or Mauritius or Mozambique or Namibia or Niger or Nigeria or Rwanda or "Sao Tome" or Senegal or Seychelles or "Sierra Leone" or Somalia or "South Africa" or Sudan or Swaziland or Eswatini or Tanzania or Togo or Uganda or Zambia or Zimbabwe))

# 9	711,668	#8 OR #7
# 10	117	#9 AND #6 AND #3 AND LANGUAGE: (English) AND DOCUMENT TYPES: (Article) Indexes=SCI-EXPANDED, SSCI, A&HCI, CPCI-S, CPCI-SSH, BKCI-S, BKCI-SSH, ESCI, CCR-EXPANDED, IC Timespan=2000-2021

OTHER SEARCHES

Databases and the search terms/phrases used to perform searches	Research Results
**AFRICAN INDEX MEDICUS:**<br>Childhood obesity and overweight among preschoolers in Titles Keywords for all material types	No eligible studies
**African Journals Online:**<br>("risk factor*" OR "determinant*" OR "predict*" OR "associate$" OR "predispose$" OR "relate$" OR "correlate$") AND ("nutritional status" OR "obesity$" OR "overweight" OR "overnutrition" OR "overnutrition" OR "overeat$" OR "overfeed$" OR "fatness" OR "bmi z score*" OR "body weight*") AND ("preschool*" OR "pre school*" OR "pre-school*" OR "kindergarten*" OR "nursery school*" OR "day care center*" OR "day care centre*" OR "daycare center*" OR "daycare centre*" OR "day-care center*" OR "day-care centre*" OR "pre-primary school*" OR "pre primary school*" OR "creche*") AND ("home*" OR "home environment*" OR "house*" OR "household*" OR "household-level" OR "mother*" OR "maternal" OR "father*" OR "paternal" OR "parent*" OR "family$" OR "caregiver*")	28 Eligible Studies
